# Activated oligoadenylate synthetase-ribonuclease L pathway promotes endothelial pyroptosis and impairs diabetic wound healing via thioredoxin-interacting protein m^6^A methylation

**DOI:** 10.1186/s43556-025-00399-9

**Published:** 2025-12-29

**Authors:** Peng Zhou, Yating Huang, Zezheng Wang, Dianxi Chen, Binbin Long, Peiliang Qin, Yiqing Li, Chao Yang, Qin Li

**Affiliations:** 1 Department of Vascular Surgery, Union Hospital, Tongji Medical College, Huazhong University of Science and Technology, Wuhan, Hubei China; 2 Department of Endocrinology, Beijing Hospital, Fifth School of Clinical Medicine, Peking University, Beijing, China; 3 Fujian Maternity and Child Health Hospital College of Clinical Medicine for Obstetrics & Gynecology and Pediatrics, Fujian, China; 4 General Surgery Department, Taihe Hospital Affiliated to Hubei University of Medicine, Shiyan, Hubei China; 5 Department of Vascular Surgery, Shandong Provincial Hospital Affiliated to Shandong First Medical University, Jinan, Shandong 250021 China

**Keywords:** OAS/RNase L, M^6A^ methylation, TXNIP, Pyroptosis, LPS, Diabetic wound

## Abstract

In diabetic skin, even in the absence of infection, the antiviral enzymes 2′-5′-oligoadenylate synthetase (OAS) and ribonuclease L (RNase L) demonstrate abnormally heightened activity. This dysregulation triggers a state of cellular stress, which not only suppresses intracellular protein synthesis but also activates innate immune responses—particularly upon pathogenic bacterial invasion. Whether the sustained activation of the OAS–RNase L pathway in diabetic skin tissue critically contributes to impaired wound healing remains to be determined. We have investigated the pyroptosis changes in human umbilical vein endothelial cells (HUVECs) treated with Lipopolysaccharide (LPS) under high glucose by RNase L small interfering RNA (siRNA) to down-regulate RNase L expression. Under high glucose conditions, we observed abnormal activation of the OAS/RNase L pathway in HUVECs, which further exacerbated cellular pyroptosis upon LPS stimulation. Abnormally activated RNase L, which reduces the expression of methyltransferase 3 (METTL3), led to decreased m^6^A methylation of thioredoxin-interacting protein mRNA (TXNIP mRNA). The decreased degradation of TXNIP mRNA by the hypomethylation leads to increased TXNIP expression, which in turn enhances pyroptosis.. In vitro experiments, the impact of RNase L inhibitor Ellagic acid (EA) on diabetic wound healing in STZ (streptozotocin)-induced diabetic mice was evaluated. We found a reduction in skin pyroptosis and improved wound healing when *EA* was administered orally. Our results demonstrate that hyperglycemia-induced OAS/RNase L activation increases endothelial cell susceptibility to pyroptosis and inflammatory responses during infection. These findings provide valuable insights for developing novel therapeutic strategies for diabetic wound management.

**Supplementary Information:**

The online version contains supplementary material available at 10.1186/s43556-025-00399-9.

## Background

Delayed wound healing in diabetes has long been a challenging clinical issue. The current consensus is that delayed healing of diabetic skin wounds is closely related to several factors: peripheral vascular disease (ischemia), neuropathy, prolonged hyperglycemic stimulation, and local infection [1]. Prolonged hyperglycemia damages endothelial cells’ integrity, leading to impaired angiogenesis, and increases the expression of pro-inflammatory factors, exacerbating the inflammatory response [2]. Diabetic wounds remain in a chronic inflammatory state and fail to progress to the proliferative phase, unlike the acute wound healing process [3].

Activating the OAS-RNase L system in the body’s antiviral defense effectively inhibits viral replication. OAS proteins were among the first double-stranded ribonucleic acid (dsRNA) sensors to be discovered. During viral infection, dsRNA activates OAS, then catalyzes the synthesis of 2′−5′-linked oligoadenylates (2-5A) from adenosinetriphosphate (ATP). Subsequently, 2-5A binds to and activates RNase L, leading to the degradation of single-stranded RNA. This process inhibits the synthesis of both cellular and viral proteins, promoting autophagy and apoptosis, thus effectively halting viral replication. Furthermore, OAS activation also stimulates the innate immune response by inducing type I interferon production [4, 5]. It also regulates the pro-inflammatory gene expression and is involved in multiple biological functions, such as autophagy, apoptosis, NOD-like receptor thermal protein domain associated protein 3 (NLRP3) inflammasome activation, cell migration, and adhesion [6–8], playing significant roles in various human diseases, including cancer, heart injury, and acute lung injury [9, 10]. Recent studies have demonstrated that the innate immune system is dysregulated in patients with type 1 diabetes, with the OAS gene being highly overexpressed in the islets of newly diagnosed type 1 diabetes patients [11]. Additionally, Wu et al. compared the transcriptomic profiles of skin tissue from type 2 diabetes patients and non-diabetic individuals using the genotype-tissue expression database. They discovered that three genes of the 2’−5’ OAS family, OAS1, OAS2, and OAS3, were significantly upregulated in type 2 diabetes patients [12], suggesting an antiviral response in diabetic cells, predisposing them to programmed cell death and triggering inflammatory reactions even in the absence of pathogens.

Patients with type 2 diabetes are at a higher risk of infection. Studies indicate that their likelihood of hospitalization due to infection is more than doubled, and the infection-related mortality rate is also higher [13, 14]. The wound healing capacity of diabetic patients is significantly compromised due to a weakened antimicrobial response. Research indicates that immune cell function in diabetic wounds is compromised, reducing the body’s ability to fight bacterial infections and rendering the wounds more susceptible to bacterial invasion [15]. Compared to ordinary wounds, diabetic wounds are more challenging to heal after infection, a phenomenon closely linked to the hyperglycemic environment and abnormal regulation of the inflammatory response [16]. Although it is believed that hyperglycemia contributes to immune dysfunction, thereby increasing the infection risk in diabetic patients, the molecular mechanisms underlying the link between blood glucose and infection remain still unclear. Investigating whether the persistent activation of the OAS-RNase L pathway in diabetic skin tissue leads to increased cell death and inflammation in response to bacterial invasion may provide new insights into the molecular mechanisms linking blood glucose and infection.

This study investigates the involvement of the OAS-RNase L pathway in endothelial cell death in diabetic wounds upon bacterial infection. We assessed pyroptosis and explored the underlying mechanisms in HUVECs under combined high-glucose and LPS stimulation following RNase L knockdown. To assess the therapeutic potential of the RNase L inhibitor EA, in impaired wound healing, we applied it to wounds in diabetic mice. This study aims to provide new insights and directions for treating delayed wound healing in diabetes.

## Results

### Increased OAS/RNase L expression in HUVECs cultures under high glucose

**Fig. 1:**
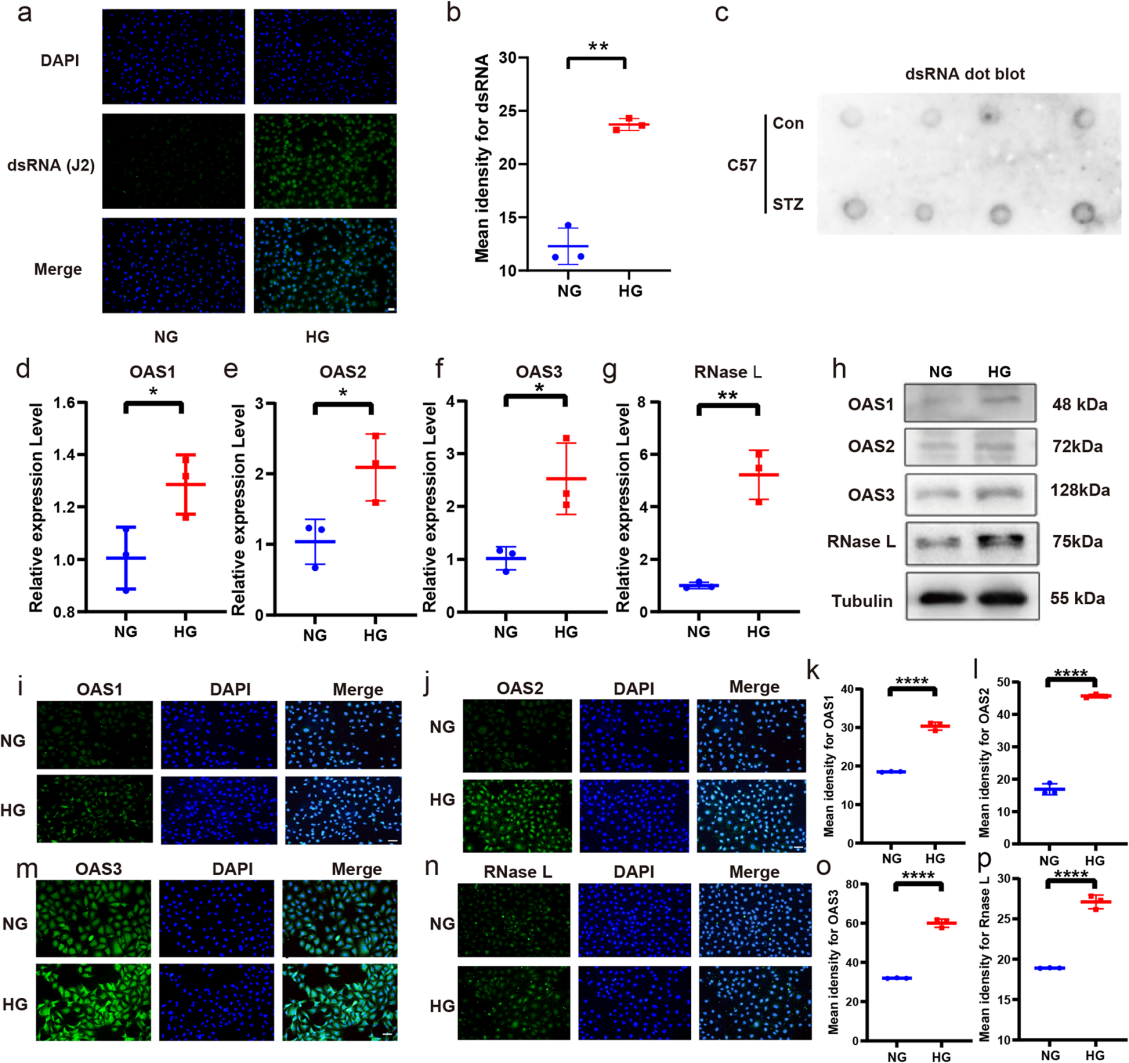
Elevated expression of OSA-RNase L in the HUVECs stimulated by high glucose. Immunofluorescence assay and semi-quantitative analysis of dsRNA (**a**-**b**). dot blot assay of dsRNA in the diabetic mouse skin (**c**). RT-qPCR assay of OAS1 mRNA (**d**), OAS2 mRNA (**e**), OAS3 mRNA (**f**), RNase L mRNA (**g**) expression in HUVECs stimulated by high glucose. Western blot assay of OAS1, OAS2, OAS3 and RNase L protein (**h**), and immunofluorescence assay and semi-quantitative analysis of OAS1, OAS2, OAS3 and RNase L (i-p). The results are presented as the mean ± SEM, *p < 0.05, **p < 0.01, ****p < 0.0001. Each experiment was replicated for thrice. The number of mice used in this experiment was four per group

To investigate how a high-glucose environment affects the OAS/RNase L pathway, we first measured dsRNA levels in HUVECs under high glucose and diabetic mouse skin tissues. Cellular immunofluorescence analysis revealed an increase in dsRNA levels (Fig. 1a-b), which was further supported by dot blot assays of mouse skin tissues (Fig. 1c). Then we analyzed the expression of OAS1, OAS2, OAS3, and RNase L in HUVECs. In the high glucose-stimulated HUVEC model, qPCR analysis revealed upregulated mRNA expression of OAS1, OAS2, OAS3, and RNase L (Fig. 1d-g). Consistent with these findings, western blot and immunofluorescence assays further confirmed the enhanced protein expression of OAS1, OAS2, OAS3 and RNase L (Fig. 1h-p).

### High glucose increases pyroptosis in HUVECs treated with LPS

**Fig. 2:**
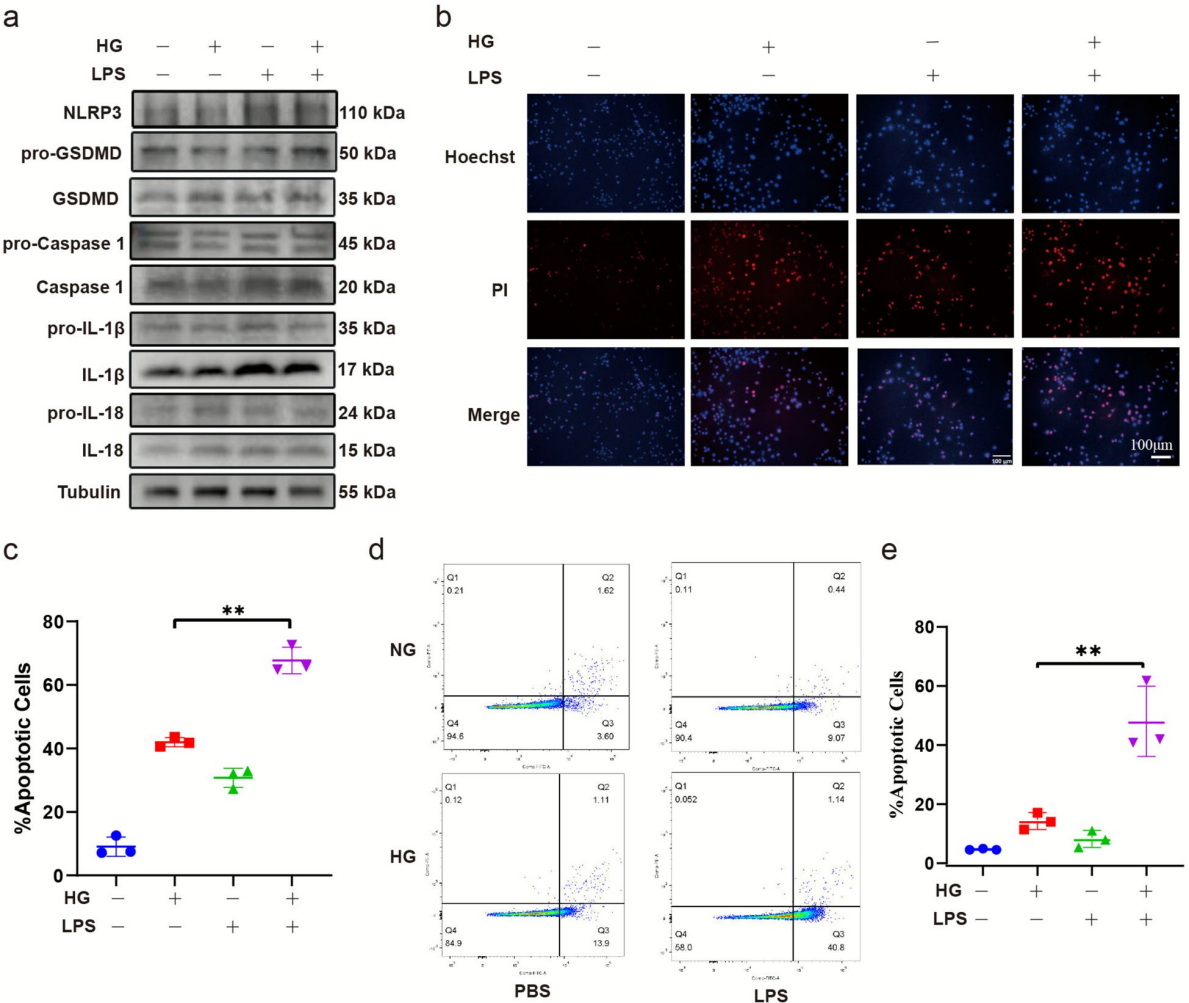
High glucose stimulation increases pyroptosis of HUVECs induced by LPS. Western blot assay of NLRP3, GSDMD, caspase-1, IL-1β and IL-18 proteins (**a**). HDMEC apoptosis was detected by Hoechst 33,342 staining (**b**). Quantification of apoptosis cells (%) (**c**). The apoptotic cells were measured by flow cytometry (**d**) and analyzed by flow Jo (**e**). The results are presented as the mean ± SEM, *p < 0.05, **p < 0.01. Each experiment was replicated for thrice

Under high glucose conditions, the increased expression of OAS/RNase L in HUVECs suggests that the cells are in an antiviral state, rendering them more susceptible to cell death, especially when exposed to external stimuli. Consequently, we assessed the occurrence of pyroptosis after stimulating HUVECs cultured in high glucose with LPS in vitro using western blot, Hoechst/PI and flow cytometry. western blot analysis showed increased expression of NLRP3, GSDMD, caspase 1, IL-1β and IL-18 proteins (Fig. 2a). Apoptotic cells were visualized using Hoechst/PI double staining (Fig. 2b-c), and annexin V–FITC/PI double staining followed by flow cytometry (Fig. 2d-e). Collectively, these results suggest that high glucose promotes pyroptosis in LPS-treated HUVECs.

### RNase-L regulated pyroptosis in HUVECs treated with LPS

**Fig. 3:**
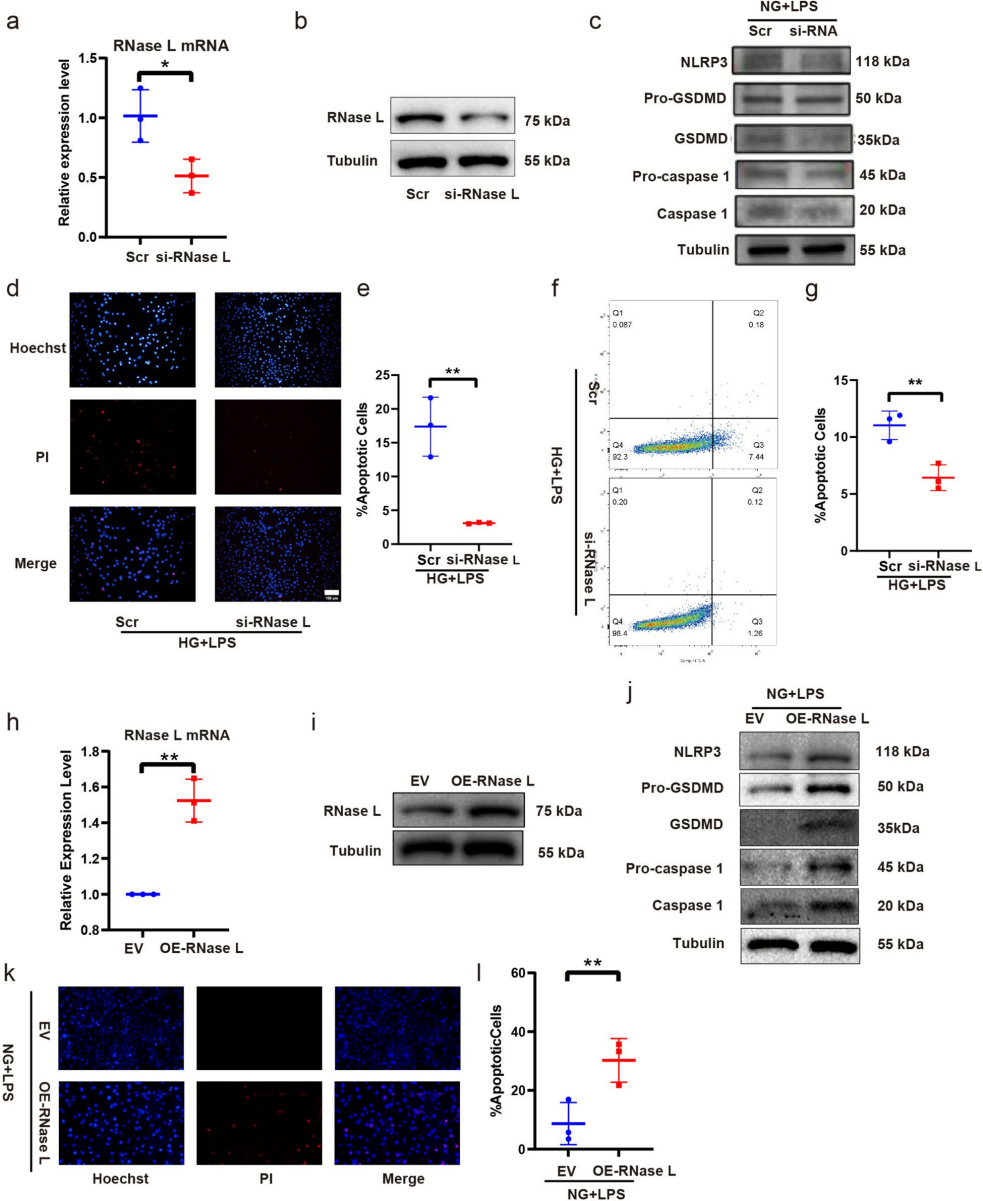
RNase-L regulated pyroptosis in HUVEC treated with LPS. qPCR and western blot to validate the knockdown efficiency of siRNA-RNase L (**a**-**b**). Western blot assay of NLRP3, GSDMD and caspase-1 proteins expression (**c**). HDMEC apoptosis was detected by Hoechst 33,342 staining (**d**). Quantification of apoptosis cells (%) (**e**). The apoptotic cells were measured by flow cytometry (**f**) and analyzed by flow Jo (**g**). qPCR and western blot to validate the overexpression efficiency of RNase L plasmid (**h**-**i**). Western blot assay of NLRP3, GSDMD and caspase-1 proteins expression (**j**). HDMEC apoptosis was detected by Hoechst 33,342 staining (**k**). Quantification of apoptosis cells (%) (**l**). The results are presented as the mean ± SEM, *p < 0.05, **p < 0.01. Each experiment was replicated for thrice

To elucidate the role of the OAS/RNase L pathway, we employed RNase L knockdown and overexpression assays to study its involvement in the LPS response of HUVECs. We knockdown RNase L expression in HUVEC through si-RNA transfection (Fig. 3a-b). Subsequently, we performed pyroptosis detection on HUVEC cells. Western blot analysis showed that the expression levels of NLRP3, GSDMD and caspase 1 were decreased in HUVECs after RNase L knockdown (Fig. 3c). Apoptosis was evaluated by Hoechst 33342/PI staining (Fig. 3d-e) and flow cytometry using FITC-conjugated Annexin V and PI (Fig. 3f-g). The results indicated that RNase L knocdown attenuated LPS-induced pyroptosis under high glucose conditions. We overexpressed RNase L via plasmid and characterized its effect pyroptosis in HUVEC cells treated with LPS under normal glucose. The validation of RNase L expression after plasmid transfection is shown in the qPCR and Western blot results in Figture 3 (Fig. 3h-i). Western blotting revealed the expression of NLRP3, GSDMD and caspase 1 increased in HUVEC following RNase L overexpression (Fig. 3j). Hoechst 33342/PI staining showed apoptosis increased (Fig. 3k-l).

### TXNIP expression was decreased in the knockdown of RNase L in HUVECs treated with LPS under high glucose by RNA sequencing (RNA-seq)

**Fig. 4:**
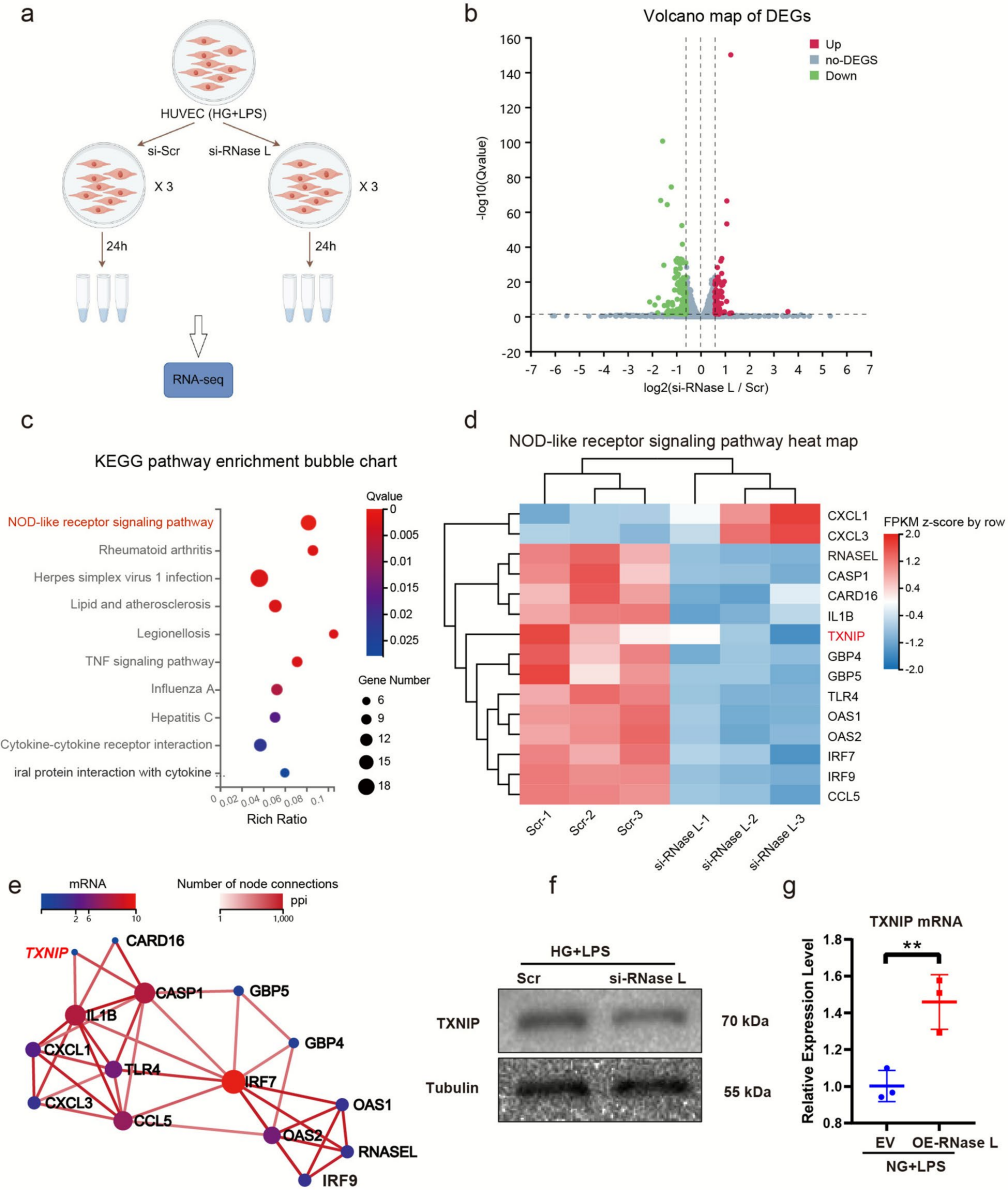
TXNIP expression was decreased in the knockdown of RNase L in HUVECs treated with LPS under high glucose by RNA-seq. Design of RNA-seq Sequencing Experiment (**a**). The volcano of DEGs in HUVESs prior and post siRNA knockdown of RNase L treated with LPS under high glucose (**b**). Top 10 KEGG pathway analysis of the identified DEGs (**c**). The heatmap of DEGs in NOD-like receptor signaling pathway which ranked first in KEGG pathway (**d**). Protein–protein interaction analysis of 15 differentially expressed functional genes in NOD-like receptor signaling pathway (**e**). Western blot assay of TXNIP protein (**f**). Analysis of TXNIP mRNA by qPCR in HUVECs upon RNase L overexpression (**g**). The results are presented as the mean ± SEM, **p < 0.01. Each experiment was replicated for thrice

We performed RNA-seq analysis to assess the role of RNase L in HUVEC treated with LPS under high glucose (Fig. 4a). The differentially expressed genes (DEGs) were presented in a volcano plot (Fig. 4b). Gene ontology (GO) enrichment analysis was performed on these DEGs across the GO categories. The top ten significantly enriched GO terms from each category are shown in Supplemental information (Fig. S1a-c). Notably, GO terms related to immune regulation and inflammatory response were prominently enriched among the DEGs. KEGG pathway enrichment analysis (Fig. 4c) revealed ten significant pathways, with the nucleotide-binding oligomerization domain (NOD)-like receptor signaling pathway being the most significant. Then we focused on a detailed analysis of its fifteen constituent target genes (Fig. 4d). A protein–protein interaction network was constructed using all DEGs. Among the nodes in this network, TXNIP emerged as a hub gene. This key protein in the cellular stress response pathway is known to primarily regulate the activation of pyroptosis (Fig. 4e). We next examined alterations in TXNIP expression. In HUVEC cells, knockdown of RNase L led to a decrease in TXNIP protein levels, as shown by western blot (Fig. 4f). Consistent with this observation, qPCR and IF analyses further confirmed the downregulation of TXNIP expression (Fig. S1d-f). Conversely, overexpression of RNase L resulted in elevated TXNIP mRNA expression (Fig. 4g). The above results suggested that RNase L-induced TXNIP overexpression caused HUVEC pyroptosis treated with LPS under high glucose.

### Pyroptosis induced by high glucose and LPS in HUVECs is mediated by the RNase L-dependent regulation of TXNIP mRNA m^6A^ methylation via METTL3

**Fig. 5:**
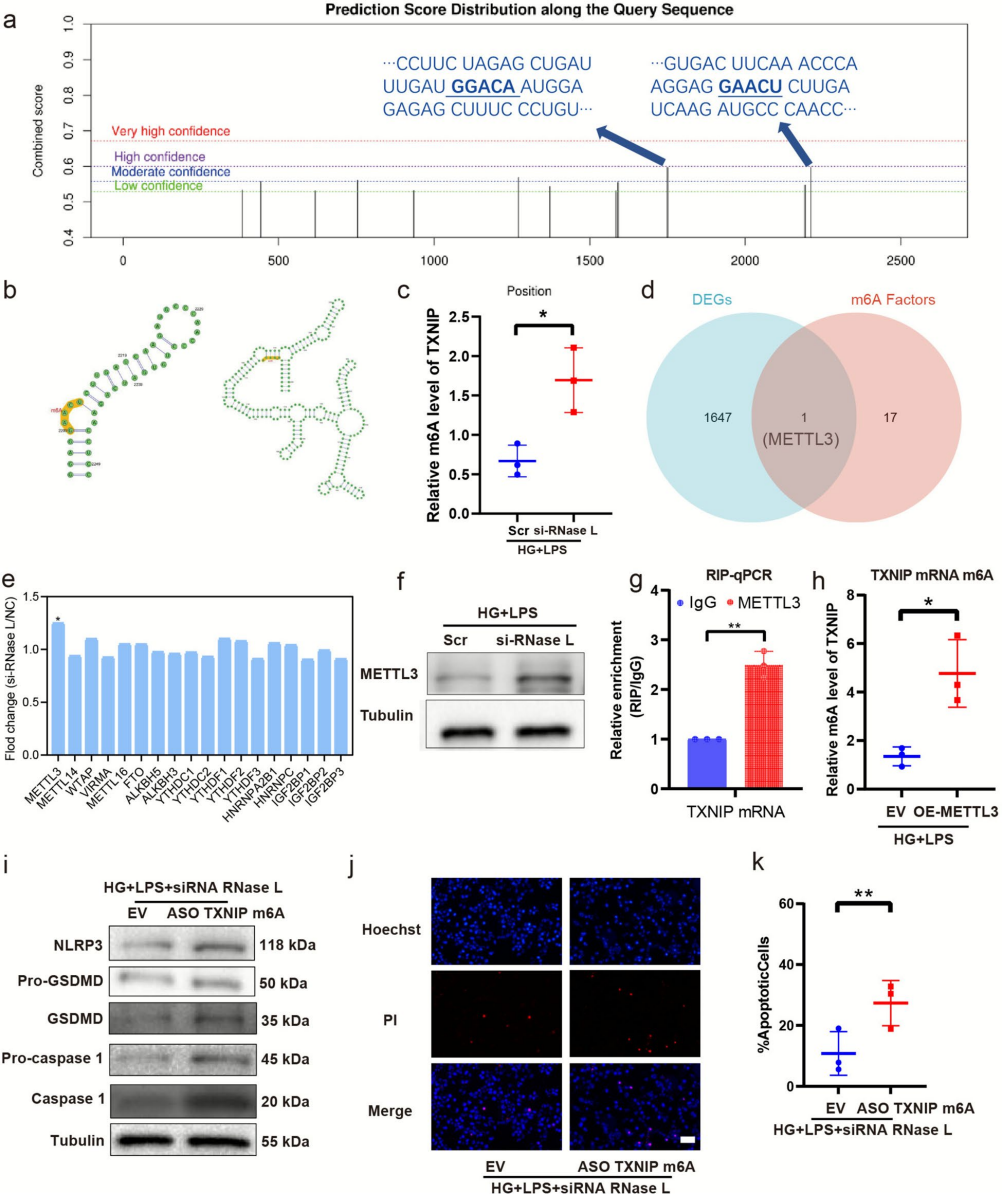
Pyroptosis induced by high glucose and LPS in HUVECs is mediated by the RNase L-dependent regulation of TXNIP mRNA m6A methylation via METTL3.. Prediction results of TXNIP mRNA in SRAMP website show the potential site of m^6^A modification. The blue arrow points sites with moderate confidence (**a**). The predicted molecular structure of the two sites on the website (**b**). MeRIP-qPCR analysis of TXNIP mRNA (**c**). Wayne diagram displaying the hub genes METTL3 between DEGs and the genes of common N6-methyladenosine (m6A) RNA methylation regulators (**d**). Distribution of fold-change of differential expression for 18 common N6-methyladenosine (m6A) RNA methylation regulators expressed genes (**e**). Western blot assay of METTL3 (**f**). RIP-qPCR assay of METTL3 with TXNIP mRNA (**g**). Analysis of TXNIP mRNA by qPCR in HUVECs upon METTL3 overexpression (**h**). Analysis of NLRP3, GSDMD and caspase-1 by western blot in HUVECs upon mutating the m^6A^ sites in TXNIP transcripts with ASO TXNIP m^6A^ (**i**). HDMEC apoptosis was detected by Hoechst 33,342 staining (**j**). Quantification of apoptosis cells (%) (**k**). The results are presented as the mean ± SEM, *p < 0.05, **p < 0.01. Each experiment was replicated for thrice

The expression of molecules is regulated by various pathways, with methylation being a key mechanism. Given the RNA cleavage function of RNase L, we aim to investigate its potential role in regulating m^6A^ methylation of TXNIP mRNA—an area that has been rarely explored. We used the SRAMP website to predict the m^6A^ modification sites in TXNIP. As shown in Fig. 5 (Fig. 5a), two potential sites were identified with moderate to high confidence, respectively. The molecular structures corresponding to these two sites were also modeled (Fig. 5b). Consequently, we conducted MeRIP-qRT-PCR experiments to confirm the modifications in m^6^A levels. The findings revealed that the m^6^A modification level of TXNIP mRNA had been increased (Fig. 5c). To further elucidate how RNase L affects the m^6A^ methylation of TXNIP mRNA, we performed an analysis of differential m^6A^ regulator expression. The results show that among the 17 genes commonly involved in m^6A^ methylation regulation, only METTL3 was found to be included in the differentially expressed genes (DEGs) (Fig. 5d–e). Four key regulatory genes—METTL3, METTL14, fat mass and obesity-associated protein (FTO), and WTAP—were selected for further validation by qPCR. Among these, only METTL3 exhibited statistically significant differential expression (Fig. S1g-j). Western blot analysis confirmed the upregulation of METTL3 expression (Fig. 5f). Similarly, IF assays also indicated a upregulation in METTL3 in Supplemental information (Fig. S1k-l).

To clarify whether METTL3 directly methylates TXNIP mRNA, we performed co-immunoprecipitation experiments (RIP) of METTL3 with TXNIP mRNA. The results clearly show that METTL3 specifically interacts with TXNIP mRNA (Fig. 5g), supporting a direct binding relationship. Subsequently, we overexpressed METTL3 in HUVECs treated with LPS under high glucose and detected an increase in the methylation level of TXNIP mRNA m^6A^ (Fig. 5h). Furthermore, to determine if mutating the m^6A^ sites in TXNIP transcripts would abolish the effects of RNase L knockdown, we examined the changes in pyroptosis after introducing site-directed mutations into the two top-scoring predicted m^6A^ sites (ASO TXNIP m^6A^). Under conditions where these m^6A^ sites were mutated, we observed an increase in pyroptosis. Western blotting revealed the expression of NLRP3, GSDMD and caspase 1 increased (Fig. 5i) and hoechst 33342/PI staining showed apoptosis increased (Fig. 5j-k). Thus, our findings confirm that pyroptosis induced by high glucose and LPS in HUVECs is mediated by the RNase L-dependent regulation of TXNIP mRNA m^6A^ methylation via METTL3.

### The combined exposure to high glucose and LPS impaired TXNIP methylation in HUVECs and thus promoted its expression

**Fig. 6:**
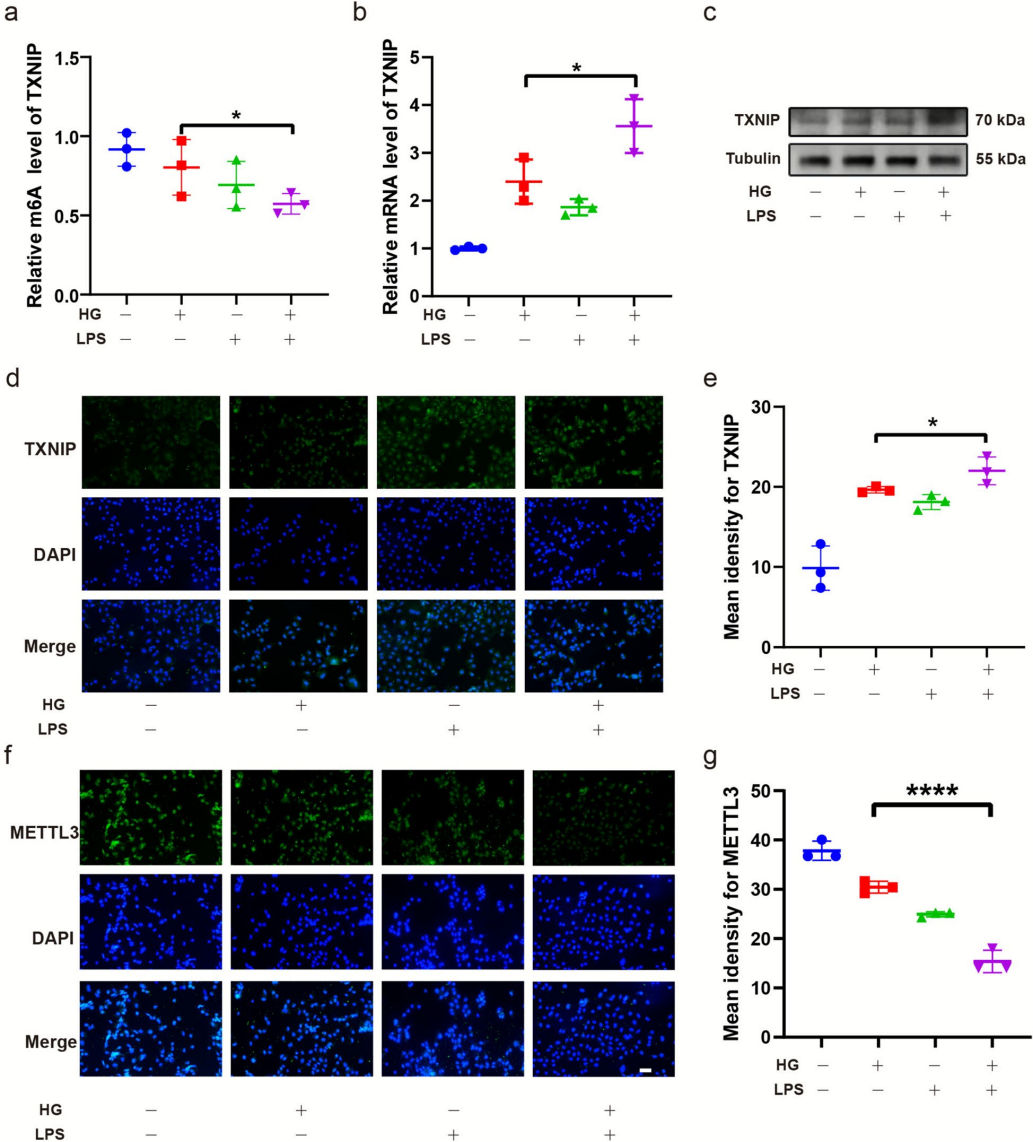
m^6^A methylation of the TXNIP mRNA was decreased and TXNIP expression increased in HUVECs treated with LPS under high glucose. MeRIP-qPCR analysis of TXNIP mRNA (**a**). RT-qPCR assay of TXNIP mRNA expression (**b**). Western blot assay of TXNIP protein (**c**), and immunofluorescence assay and semi-quantitative analysis of TXNIP (**d**-**e**). Immunofluorescence assay and semi-quantitative analysis of METTL3 (**f**-**g**). The results are presented as the mean ± SEM, *p < 0.05, ****p < 0.0001. Each experiment was replicated for thrice

To determine whether high glucose influences LPS-treated HUVEC cells through TXNIP, we assessed TXNIP expression in cultured cells. First, we evaluated the m^6A^ methylation status of TXNIP. MeRIP-qRT-PCR results revealed that TXNIP exhibited a relatively lower methylation level in HUVECs compared with cells cultured under normal glucose conditions (Fig. 6a). Subsequently, we analyzed TXNIP mRNA and protein levels. qPCR analysis indicated elevated TXNIP mRNA expression (Fig. 6b), while western blotting and immunohistochemistry (IHC) consistently confirmed increased protein expression (Fig. 6c–e). In addition, METTL3 protein expression was assessed by IHC, which showed a decrease under high glucose conditions (Fig. 6f–g).

### The RNase L inhibitor EA reduced pyroptosis and promoted angiogenesis in HUVECs

**Fig. 7:**
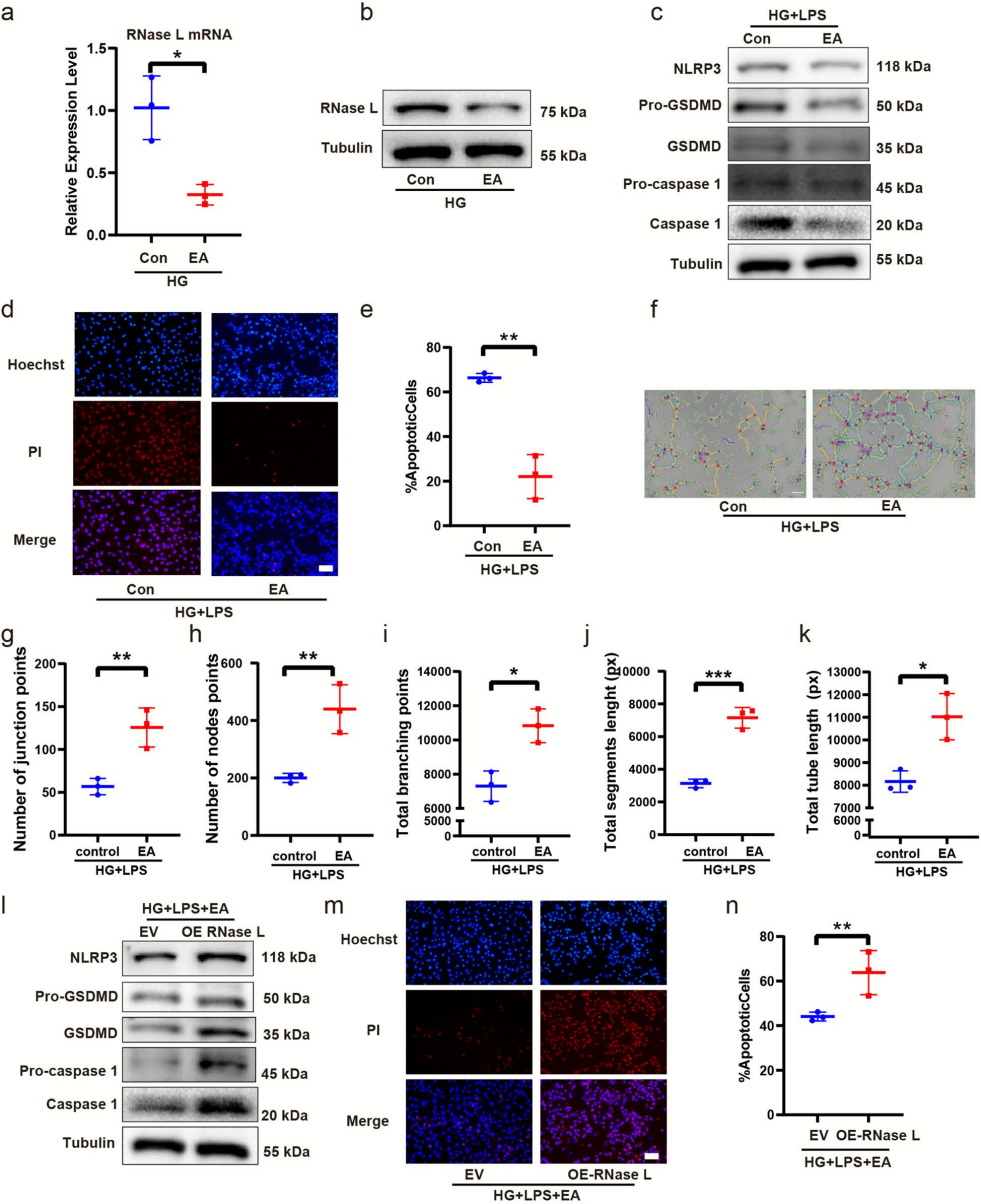
The RNase L inhibitor EA reduced pyroptosis and promoted angiogenesis in HUVECs. RT-qPCR assay of RNase L mRNA (**a**) expression in HUVECs stimulated by high glucose. Western blot assay of RNase L protein (**b**). Western blot assay of NLRP3, GSDMD and caspase-1 proteins expression (**c**) in HUVECs treatment with EA under high glucose. HDMEC apoptosis was detected by Hoechst 33,342 staining (**d**). Quantification of apoptosis cells (%) (**e**). Tube formation assay of HUVECs (**f**) and result analysis by number of junction points (**g**), number of nodes points (**h**), total branching points (**i**), total segments length (**j**), and total tube length (**k**). Analysis of NLRP3, GSDMD and caspase-1 by western blot in HUVECs upon RNase L overexpression followed by EA treatment under stimulation of LPS and high glucose (**l**). HDMEC apoptosis was detected by Hoechst 33,342 staining (**m**). Quantification of apoptosis cells (%) (**n**). Each experiment was replicated for thrice

To further establish the functional link between EA and RNase L, we examined whether EA treatment reverses RNase L-associated pyroptosis. Firstly, we evaluated whether EA affects RNase L expression. The results showed that in HUVECs under high-glucose conditions, EA treatment reduced RNase L expression (Fig. 7a-b). In HUVECs subjected to high glucose and LPS stimulation, EA administration attenuated cellular pyroptosis. Western blotting revealed the expression of NLRP3, GSDM and caspase 1 decreased (Fig. 7c) and hoechst 33342/PI staining (Fig. 7d-e) showed apoptosis decreased. Furthermore, we have performed an in vitro endothelial tube formation assay using HUVECs to functionally assess angiogenesis. HUVECs were cultured under high glucose conditions and stimulated with LPS to create a dysfunctional cellular model. The results show that treatment with EA promoted tube formation compared to the control group (Fig. 7f-k).

To verify whether the role of EA is mediated by its inhibition of RNase L, we have performed a rescue experiment. Specifically, we overexpressed RNase L in HUVECs via plasmid transfection. These cells were then stimulated with LPS under high-glucose conditions, followed by treatment with EA. We subsequently examined the changes in pyroptosis. Western blotting revealed the expression of NLRP3, GSDMD and caspase 1 increased (Fig. 7l) and hoechst 33342/PI staining showed apoptosis increased compared to control group (Fig. 7m-n). The results demonstrated that overexpression of RNase L effectively reversed the suppressive effect of EA on pyroptosis, indicating that the action of EA is dependent on its inhibition of RNase L.

### Oral gavage of EA accelerated diabetic mice wound healing and promoted angiogenesis dependent on its inhibition of RNase L

**Fig. 8:**
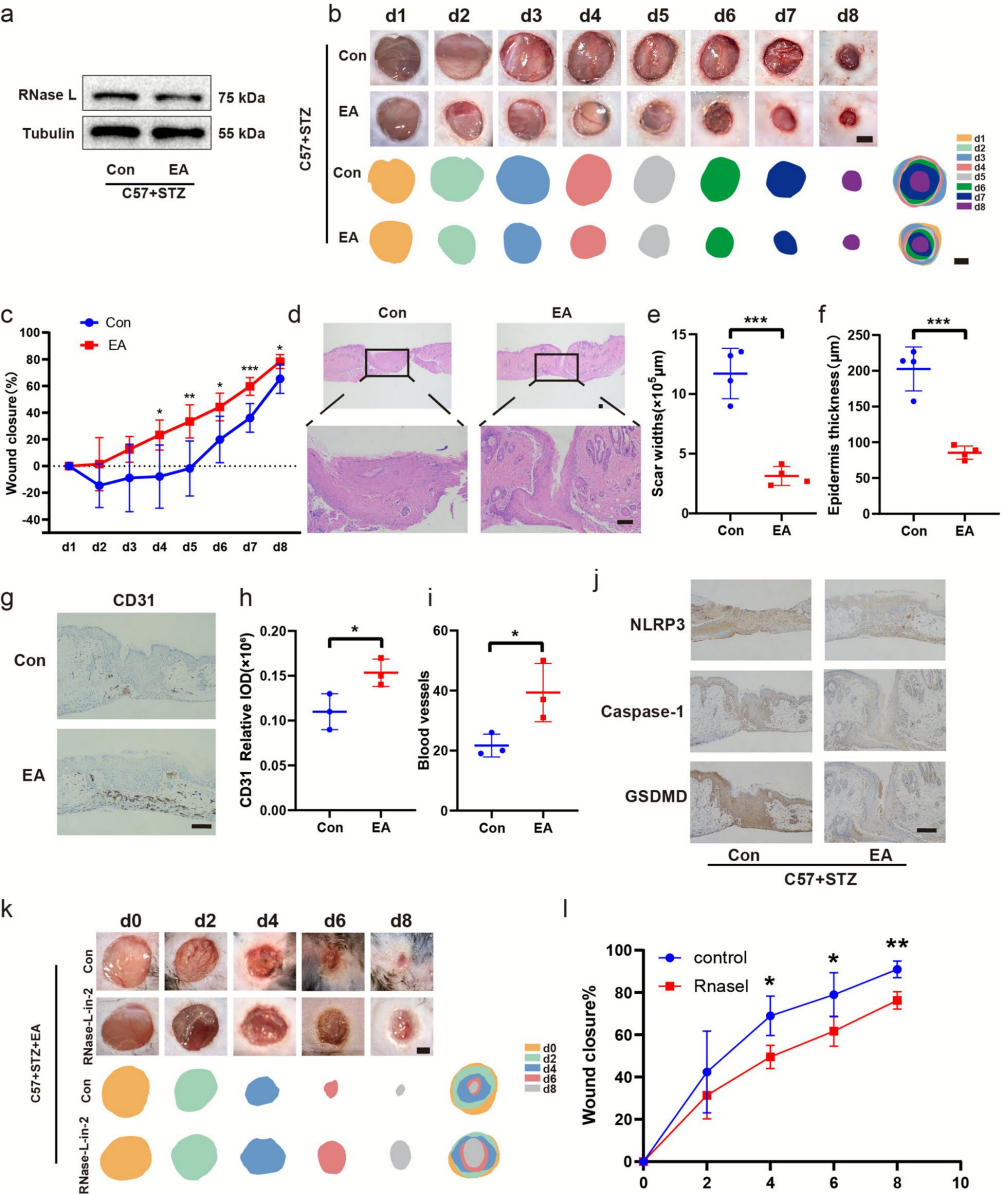
Oral gavage of EA accelerated diabetic mice wound healing and promoted angiogenesis dependent on its inhibition of RNase L Western blot assay of RNase L expression (**a**) in diabetic mouse skin after oral gavage of EA. Representative gross images of diabetic wounds in mice at day 1 to 8 (**b**). Wound healing rates in each group at different time points (**c**). H&E staining of diabetic wound at day 8 (**d**). Widths of scars (**e**) and thickness (**f**) of the epidermis. Representative immunohistochemical CD31 staining images (**g**) and relative IOD of CD31 expressions (**h**) of mice wounds in different treatment groups. The quantification of CD31-stained blood vessels in the wound sites (**i**). Analysis of NLRP3, GSDMD and caspase-1 by western blot (**j**). Representative gross images of diabetic wounds in mice applied RNase L-specific agonist (RNase L-in-2) topically to the wound following oral administration of EA (**k**). Wound healing rates in each group at different time points (**l**). The results are presented as the mean ± SEM, *p < 0.05, **p < 0.01, ***p < 0.001. The number of mice used in this experiment was two per group, and two identical wounds were created on each mouse

To investigate the role of RNase L in diabetic wound healing, we examined the therapeutic potential of EA, a potent natural compound recently identified as an inhibitor of RNase L. Firstly, we evaluated the affects of EA on RNase L expression in diabetic mouse skin. The results showed EA treatment reduced RNase L expression (Fig. 8a). Subsequently, we observed the wound healing status after EA treatment. The results demonstrated that EA treatment significantly enhanced the wound healing rate compared to the saline control (Fig. 8b). To quantitatively compare healing among groups, wound closure rates were calculated and statistical analysis indicated that from day four onward, the EA-treated group showed a significantly higher rate of wound closure relative to the control (Fig. 8c). To validate wound width measurements, we examined histological sections of the wound area. Hematoxylin and eosin (H&E) staining demonstrated narrower scars in the EA-treated group compared with the control group (Fig. 8d-e). In addition, EA treatment improved the thickness of re-epithelialization within the scar tissue (Fig. 8f). We also evaluated angiogenesis and observed a marked increase in CD31-positive cells in EA-treated wounds (Fig. 8g). Quantitative analysis confirmed a higher number of CD31-positive cells and blood vessel formationin the EA group relative to the control group (Fig. 8h-i). Subsequently, we evaluated the effect of EA treatment on pyroptosis in skin tissues from the wound areas of diabetic mice. IHC analysis was performed to assess the expression of NLRP3, GSDMD and caspase-1 (Fig. 8j), and quantitative analysis of IHC results (Fig. S1m-o). The results showed that the EA group exhibited reduced levels of pyroptosis compared with the control group. As an reviewer suggested, we have included an additional animal experiment group in which normal mice (intraperitoneal injection of STZ solvent citrate buffer) were administered EA and monitored for wound healing. The results showed no significant difference in wound healing compared to the control group (Fig. S2a-c).

Given the multi-target nature of EA, we performed additional animal experiments in which an RNase L-specific agonist (RNase L-in-2) was applied topically to the wound following oral administration of EA in mice, to more directly evaluate the role of EA in diabetic wound healing. We observed delayed wound healing in this group (Fig. 8k-l), indicating that activation of RNase L can counteract the beneficial effects of EA.

## Discussion

Recently, research has focused on the antiviral activity of OAS proteins and the OAS/RNase L pathway. Unlike exogenous dsRNA, endogenous dsRNA is produced during physiological processes within cells, mainly arising from introns and inverted intergenic repeat elements [17]. Moreover, studies have demonstrated that endogenous dsRNA can accumulate when mechanisms maintaining DNA or RNA integrity are disrupted, such as the absence of ADAR1 or DNA methylation. These endogenous dsRNA molecules can activate the OAS-RNase L pathway, triggering an antiviral response [18]. Our research revealed that OAS1, OAS2, OAS3, and RNase L expression significantly increased in high-glucose HDMECs under high-glucose stimulation, suggesting that in a high-glucose environment, endogenous dsRNA accumulates, promoting the activation of the OAS-RNase L pathway. Consequently, a high-glucose environment affects the cell’s metabolic state and may also influence RNA stability, producing endogenous dsRNA and establishing an antiviral state in a cell. This increase in cellular fragility could represent a novel molecular basis for the delayed wound healing observed in diabetes.

Pyroptosis is programmed cell death triggered by inflammation, characterized primarily by cell membrane rupture and the release of intracellular contents. This phenomenon induces an inflammatory response in surrounding tissues and is critical in various pathological conditions, particularly infection and inflammation [19, 20]. Pyroptosis impairs cellular function and modifies their response to inflammatory factors in vascular endothelial cells, affecting vascular function. Studies have demonstrated that hyperglycemia-induced pyroptosis in vascular endothelial cells contributes significantly to diabetic microvascular complications, including diabetic retinopathy, diabetic nephropathy, and atherosclerosis [21]. This study discovered that high glucose conditions combined with LPS stimulation led to a marked increase in pyroptosis in HDMECs. However, the level of pyroptosis in HDMECs was significantly reduced after RNase L expression was modulated, highlighting the importance of RNase L in regulating pyroptosis. We observed significant changes in inflammatory signaling pathways in HDMECs following RNase L intervention under LPS stimulation through RNA-seq analysis. Notably, the NOD-like receptor signaling pathway exhibited the most prominent changes, with most of the enriched DEGs being downregulated, suggesting suppression of this pathway, indicating that RNase L can mediate the immune response induced by LPS, mitigating the inflammatory response through the regulation of the NOD-like receptor signaling pathway. Some studies suggest that RNase L mediates the LPS-induced immune response, regardless of its nuclease activity. For instance, RNase L modulates macrophage function by regulating the pro-inflammatory and anti-inflammatory gene expression following LPS stimulation of macrophages [22]. Although our study demonstrated that LPS stimulation increases pyroptosis in HDMECs under high glucose conditions and is associated with RNase L activation, the specific molecular regulatory mechanisms involved require further investigation.

Methylation is an important aspect of epigenetic modification and is crucial in the study of delayed wound healing in diabetes. Recent research has demonstrated a substantial correlation between delayed wound healing in diabetic patients and changes in methylation status [23–25]. It remains unclear whether RNase L, an endoribonuclease, regulates DNA or RNA degradation by m^6^A methylation. Recently, Park et al. discovered that linear and circular RNAs containing m^6^A can undergo endoribonucleolytic cleavage through the YTHDF2-HRSP12-RNase P/MRP axis [26]. Zhang et al. have demonstrated that ARID1A initiates m^6^A modification of R-loopsare by recruiting METTL3 to local double-strand breaks, which in turn recruit RNase H1 to promote the degradation of R-loopsare, thereby ensuring genome stability [27]. These findings reveal RNases’ function in regulating gene expression by m^6^A methylation. However, the specific regulatory mechanisms, particularly the role and function of RNase L, require further investigation. Interestingly, in our study, the changes in METTL3 expression were particularly notable among several molecules related to methylation regulation after interfering with RNase L expression in HDMEC cells. This finding suggests that RNase L may not only participate in RNA degradation but also regulate methylation by modulating the levels of METTL3 expression. METTL3’s activity, an important m^6^A methyltransferase, directly affects the methylation status of RNA, thereby influencing RNA stability and biological functionality [28]. This result offers a novel perspective on the biological function of RNase L, suggesting that it regulates the methylation process and may delay wound healing in diabetes.

TXNIP is one of the most significantly upregulated genes in human pancreatic islet cells when exposed to high glucose levels, primarily regulating glucose homeostasis by inducing β-cell apoptosis [29]. TXNIP elevated levels are found in patients with diabetes and chronic hyperglycemia, and this is closely linked to the onset of diabetes and its complications [30]. TXNIP directly interacts with the major antioxidant protein thioredoxin, inhibiting its antioxidant function and expression, thus controlling cellular redox signaling and maintaining redox balance [31]. Besides, TXNIP regulates endoplasmic reticulum stress-mediated NLRP3 inflammasome complex formation, which triggers mitochondrial stress, leading to apoptosis and promoting pyroptosis [32, 33]. Consequently, regulation of TXNIP expression plays a crucial role in cellular function. Our study discovered that TXNIP expression could be modulated by m^6^A methylation, and RNase L can reduce TXNIP m^6^A methylation by downregulating MELLT3 expression. The m^6^A modification mediates gene expression in various ways, such as RNA degradation, splicing, and nuclear export. The FTO Knockdown increases the amount of m^6^A in mRNA. Concurrently, the ALKBH5 gene deletion significantly reduces the cytoplasmic mRNA level, indicating that the activity of these two demethylases significantly influences mRNA export and RNA metabolism [34]. “Readers” are a group of m^6^A-binding proteins, including YTHDC1, YTHDC2, and YTHDF1-3, which specifically recognize and mediate the biological functions of m6A-modified RNA. For instance, YTHDF2 selectively binds to m^6^A-modified transcripts and directs the complex to cellular RNA decay sites, thereby accelerating the degradation of m^6^A-modified transcripts [35]. Recent studies have demonstrated that the absence of the demethylase FTO in LPS-induced acute lung injury alleviates TXNIP/NLRP3 activation-mediated pyroptosis in alveolar epithelial cells triggered by LPS [9]. Although the authors could not determine the role of FTO in regulating TXNIP expression, the absence of FTO may reduce TXNIP expression by decreasing its m^6^A methylation. In our study, the reduced level of TXNIP mRNA might be related to some m6A-modified factors, leading to increased degradation of m^6^A-modified transcripts after the increase in TXNIP m^6^A methylation; however, it requires further research to confirm it.

RNase L is a promising drug target. It plays a crucial role in antiviral innate immunity and is involved in other cellular activities. Research indicates that RNase L is associated with acute lung injury, acute ischemic heart damage, prostate cancer, colorectal cancer, and breast cancer [36], demonstrating that the role of OAS-RNase L extends beyond antiviral responses. These findings highlight the therapeutic potential of small-molecule RNase L inhibitors. Furthermore, in adenosine deaminase ADAR1 deficiency cases, self-dsRNA can activate OAS-RNase L without viral infection, leading to Aicardi-Goutières syndrome (AGS) [37]. AGS is a severe neurodevelopmental and inflammatory genetic disorder in children for which there are currently no effective drug therapies. Inhibiting RNase L exhibits potential for treating AGS. However, effective RNase L inhibitors’ development remains in its early stages, primarily due to a lack of effective tool compounds [38]. Salima and colleagues conducted a biochemical screening of a structurally diverse library of protein kinase inhibitors to search for small-molecule RNase L inhibitors. They identified EA as a promising natural compound inhibitor of RNase L [39]. EA, a natural phenolic antioxidant found in many fruits and vegetables, is commonly employed as a supplement for cancer, heart disease prevention, and other health benefits. Our research revealed that EA promotes wound healing in diabetic mice, reduces pyroptosis in wound cells, and increases the m^6^A methylation level of TXNIP, suggesting that EA plays an important role in regulating cell death, reducing inflammation, and promoting m^6^A methylation. More importantly, EA has the potential to be a therapeutic approach for delayed wound healing in diabetes, offering new treatment strategies to address this clinical challenge.

This study revealed that the expression levels of OAS1, OAS2, OAS3, and RNase L are elevated under high glucose conditions, however, the underlying molecular regulatory mechanisms remain to be fully elucidated. It is still unclear whether the high glucose environment directly induces an increase in endogenous dsRNA or whether these alterations represent secondary effects of cellular adaptation to metabolic stress. Therefore, future studies should aim to experimentally clarify the relationship between diabetic hyperglycemia and endogenous dsRNA, along with its associated pathways. Moreover, we found that RNase L can modulate the expression of multiple methylation-related factors, with a notable reduction in MELLT3 expression. Nevertheless, further investigation is needed to explore how changes in methylation status affect the expression and function of key molecules such as TXNIP. We believe that addressing these questions will enhance our understanding of the molecular mechanisms underlying impaired wound healing in diabetes and may help identify novel potential targets for clinical intervention.

High glucose conditions result in abnormal activation of OAS/RNase L in vascular endothelial cells, which increases pyroptosis easier when were invaded by bacteria. Targeting RNase L could provide a novel approach to the treatment of diabetic wound.

## Materials and methods

### Cell culture and treatment

HUVECs were obtained from the Cell Bank of the Chinese Academy of Sciences (Shanghai, China). Authentication of all cell lines was performed via short tandem repeat (STR) profiling, and mycoplasma contamination tests returned negative results. Cells were maintained at 37 °C in a humidified atmosphere of 95% air and 5% CO₂. For routine culture, MEM containing 5.5 mM glucose (#PM150220, Procell Life Science & Technology) was supplemented with 10% fetal bovine serum (FBS, #164210, Procell) and 1% antibiotics. To mimic diabetic conditions, a high-glucose medium (22.5 mM glucose, #PM150210, Procell) was utilized. Inflammatory stimulation was achieved by adding lipopolysaccharide (LPS) at a concentration of 0.1 mg/mL, with cells harvested after 24 h of LPS exposure. Cellular RNA extraction was carried out following a standard TRIzol-based procedure using RNAiso Plus (Takara, Cat # 9108). Proteins were isolated from cells using RIPA lysis buffer (Beyotime, Cat # P0013K). For immunohistochemical analysis, cells were grown on 24-mm cover slips (Solarbio, Cat # YA0352).

### Real-time quantitative PCR

The extracted RNA was reverse-transcribed into cDNA using kits (Cat # RR037A, Takara). Real-time quantitative PCR was implemented on an ABI StepOne Plus System (Applied Biosystems, Foster City, CA) using SYBR Premix Ex Taq (Cat # RR420A, Takara). Primer sequences are described in Supplementary information (Table S1). The qPCR reactions were performed in a 20 µL reaction volume containing 10 µL of 2X SYBR Green Master Mix, 0.5 µM of each forward and reverse primer, and 50 ng of cDNA template. The amplification protocol was carried out on a Instrument Model as follows: initial denaturation at 95 °C for 10 min, followed by 40 cycles of denaturation at 95 °C for 15 s and annealing/extension at 60 °C for 1 min. A melt curve analysis was subsequently performed to confirm the specificity of the amplification.

### Western blot

Proteins were resolved on 10% SDS–polyacrylamide gels and subsequently transferred to nitrocellulose membranes. The membranes were incubated overnight at 4 °C with specific primary antibodies: OAS1 (14955-1-AP), OAS2 (19279-1-AP), OAS3 (21915-1-AP), RNase L (22577-1-AP), NLRP3 (19771-1-AP), GSDMD (20770-1-AP), IL-1β (16806-1-AP), IL-18 (10663-1-AP), METTL3 (15073-1-AP), TXNIP (18243-1-AP), and Tubulin (10094-1-AP), all from Proteintech (Wuhan, China). Following primary antibody incubation, the blots were treated with an HRP-conjugated secondary antibody for 2 h at 37°C. Signal detection was performed using enhanced chemiluminescence reagent (MA0186, Meilunbio), and band intensities were quantified with ImageJ software (NIH, USA).

### dsRNA dot blot

Total RNA was spotted directly onto a nylon membrane using a dot blot apparatus. The membrane was UV cross-linked and then blocked. For dsRNA detection, the membrane was incubated with a specific primary antibody against dsRNA (J2 monoclonal antibody), followed by a horseradish peroxidase (HRP)-conjugated secondary antibody. Finally, the signal was visualized using an enhanced chemiluminescence (ECL) substrate and imaged with a chemiluminescence detection system.

### Cell immunohistochemistry

Coverslips were washed with TBS, fixed with 4% paraformaldehyde for 15 min, and permeabilized with 0.1% Triton X‐100 for 10 min. The coverslips were treated with the primary and secondary antibodies (Cat # ANT058, antGene) and then visualized with DAB (Dako) and counterstained with hematoxylin, before being analyzed by light microscopy.

### Flow cytometry apoptosis assay

Cell apoptosis was examined with a commercial kit (#C1062L, Beyotime) following the prescribed protocol. HUVECs were detached with trypsin solution devoid of EDTA (0.25% trypsin) and washed once with PBS. Cells were then resuspended in 100μL staining buffer containing 5 μL Annexin V-FITC and 5 μL PI. After incubating for 20 min in the dark at room temperature, fluorescence signals were detected with a flow cytometry. FlowJo v10.6.2 was used to analyze the results.

### siRNA and plasmid transfection

siRNA targeting the human RNase L gene (si-RNase L) and scramble siRNA (NC) were designed and synthesized by GenePharma (Shanghai, China) with specific sequence: si-RNase L: CGAAGATGTTGACCTGGTC; NC: UUCUCCGAACGUGUCACGUTT. Transient transfection was carried out with Lipo8000 (#C0533, Beyotime), following the protocols provided by the manufacturer.

### RNA sequencing

RNA sequencing and subsequent analyses were conducted by BGI (Wuhan, China; service ID # F22FTSCCKF14445). Total RNA was extracted and quality-checked (Table S2), followed by mRNA enrichment using Oligo(dT) beads for library preparation. Qualified libraries were sequenced, generating raw reads that were converted into FASTQ format. Quality control and adapter trimming of raw reads were performed with Fastp to obtain clean reads, which were then aligned to the reference genome. Downstream analyses, including gene expression quantification and further in-depth investigations, were carried out based on the aligned data. The sequencing data have been deposited in the CNGB Nucleotide Sequence Archive under accession number CNP0006423.

### Functional enrichment analysis

Differential expression analysis was performed on normalized count data utilizing the DEGseq2 package within the Dr. Tom bioinformatics platform (BGI, Shenzhen, China). Genes with a false discovery rate (FDR) q-value ≤ 0.05 and a fold change ≥ 1.5 were identified as differentially expressed (DEGs). These DEGs subsequently underwent functional enrichment analysis for Gene Ontology (GO) terms and KEGG pathways, also conducted on the Dr. Tom platform. Results from the enrichment analyses were visualized using bubble plots.

### m^6^A modification sites predicted

In this study, m6A modification sites on TXNIP mRNA were predicted using SRAMP (sequence‑based RNA adenosine methylation site predictor), a dedicated database for identifying m6A sites in mammalian RNA sequences. SRAMP relies exclusively on sequence information and does not require additional omics data, offering a streamlined approach for m6A site analysis. Predictions were carried out through the SRAMP web server (http://www.cuilab.cn/sramp) by submitting the TXNIP RNA sequence under default parameters. The output includes predicted m6A motifs (highlighted in blue) along with corresponding confidence scores. By selecting the option to visualize the highest‑confidence prediction, the associated RNA secondary structure positions can be displayed.

### Methylation detection

The total RNA was extracted and processed by methylation assay kit (Base Catalog # P-9018) according to the instructions. Briefly, the RNA was quantified and immunoprecipitated with antim6A antibody. Afterwards, reverse transcription PCR and qPCR were performed.

### RIP-qPCR

The RIP experiment was performed using the Magna RIP™ Kit according to the manufacturer’s instructions. Briefly, cell lysates were incubated with a specific antibody against the target protein or control IgG-bound magnetic beads. After extensive washing, the co-precipitated RNA was isolated and purified. Subsequently, the extracted RNA was reverse-transcribed into cDNA. The enrichment of specific RNAs was quantified by qPCR using SYBR Green, with data normalized to the Input control and presented as % of Input.

### Mutation of m^6A^ sites in TXNIP transcripts

*We performed site-directed mutagenesis on the two highest-scoring predicted m6A sites within TXNIP transcripts, and custom synthesized the sequence (5' to 3') by Sangon Biotech (sg859, shanghai, China) is as follows:(LNA-T)(LNA-A)(LNA-A)(dG)(dA)(dA)(dT)(dC)(dA)(dT)(dC)(dT)(dG)(dA)(dA)(dA)(LNA-A)(LNA-G)(LNA-C).*

### Animal and experimental design

All animal procedures were conducted in accordance with the Guide for the Care and Use of Laboratory Animals (NIH Publications No. 8023, revised 1978), and the reporting of animal experiments follows the ARRIVE guidelines 2.0. A diabetic mouse model was established using a combination of high-fat diet feeding and streptozotocin (STZ) injection. Specifically, after 4 weeks on a high-fat diet, the mice were fasted for 12 h and then administered STZ (120 mg/kg; Cat # S1312, Selleck) intraperitoneally. Blood glucose levels in non-fasted mice were monitored every 5 days. A full-thickness round skin wound model with a diameter of 0.6 cm was created on the dorsal vertebral area. The mice were then treated daily via oral gavage with either EA (40 mg/kg body weight; Cat # CM00233, Proteintech, Wuhan, China) or vehicle control for up to 8 days. Wound healing was documented daily (see Fig. S3). To more directly evaluate the role of RNase L in diabetic wound healing, the RNase L-specific agonist RNase L-in-2 (3 μg once daily, HY-121834, MCE, USA) was administered to diabetic mice with wounds that had been treated with EA. After the treatment period, the mice were euthanized, and dorsal skin tissues were collected for further analysis.

### Statistical analyses

Data are presented as mean ± SEM. Comparisons between parametric and non-parametric variables were performed with the Student’s t-test and the Mann–Whitney U test, respectively. For multi-group comparisons, one-way ANOVA followed by the least significant difference (LSD) post hoc test was applied. A *p*-value below 0.05 was considered statistically significant. All graphical representations were prepared using GraphPad Prism 9.0 and Adobe Illustrator CC 2015.

## Supplementary Information

Supplementary Material 1.

## Data Availability

The FASTQ data generated in this study have been deposited in the CNGB Nucleotide Sequence Archive (CNSA) under accession number CNP0006423 (https://db.cngb.org/data_resources/?query=CNP0006423). Additional datasets used and/or analyzed are available from the corresponding author upon reasonable request. Please contact Qin Li at li_qin@hust.edu.cn.

## Abbreviations

OAS: 2′-5′-Oligoadenylate synthetase
RNase L: Ribonuclease L
HUVECs: Human umbilical vein endothelial cells
LPS: Lipopolysaccharide
siRNA: Small interfering RNA
TXNIP mRNA: Thioredoxin-interacting protein mRNA
ATP: Adenosinetriphosphate
NLRP3: NOD-like receptor thermal protein domain associated protein 3
SRAMP database: Sequence-based RNA adenosine methylation site predictor
EA: Ellagic acid
METTL3: Methyltransferase 3
PRRs: Pattern recognition receptors
dsRNA: Double-stranded ribonucleic acid
2-5A: 2′-5′-Linked oligoadenylates
DEGs: Differentially expressed genes
