# Supplementary material for: Activated oligoadenylate synthetase-ribonuclease L pathway promotes endothelial pyroptosis and impairs diabetic wound healing via thioredoxin-interacting protein m6A methylation
Source: Mol Biomed. 2025 Dec 29;6:150. doi: 10.1186/s43556-025-00399-9 (PMC12745338; doi:10.1186/s43556-025-00399-9)
Supplement: Supplementary file 1 — Supplementary Material 1. [file 43556_2025_399_MOESM1_ESM.docx]

Activated oligoadenylate synthetase-ribonuclease L pathway promotes endothelial pyroptosis and impairs diabetic wound healing via thioredoxin-interacting protein m^6^A methylation

Peng Zhou^1#^, Yating Huang^1^^#^, Zezheng Wang^1#^, Dianxi Chen^2^, Binbin Long^3^, Peiliang Qin^4^, Yiqing Li^1*^, Chao Yang^1*^, Qin Li^1*^

^1^Department of Vascular Surgery, Union Hospital, Tongji Medical College, Huazhong University of Science and Technology, Wuhan, Hubei, China

^2^Fujian Maternity and Child Health Hospital College of Clinical Medicine for Obstetrics & Gynecology and Pediatrics, Fujian, China

^3^General Surgery Department, Taihe hospital affiliated to Hubei University of Medicine, Shiyan, Hubei, China

^4^Department of Vascular Surgery, Shandong Provincial Hospital Affiliated to Shandong First Medical University, Jinan, Shandong 250021, China

# These authors have contributed equally to this work

Correspondence：

Yi-Qing Li, Department of Vascular Surgery, Union Hospital, Tongji Medical College, Huazhong University of Science and Technology, Wuhan, Hubei, China, [yiqingli_uh@126.com](mailto:yiqingli_uh@126.com)

Chao Yang, Department of Vascular Surgery, Union Hospital, Tongji Medical College, Huazhong University of Science and Technology, Wuhan, Hubei, China,

[ychao@hust.edu.cn](mailto:ychao@hust.edu.cn)

Qin Li, Department of Vascular Surgery, Union Hospital, Tongji Medical College, Huazhong University of Science and Technology, Wuhan, Hubei, China,

[li_qin@hust.edu.cn](mailto:li_qin@hust.edu.cn)


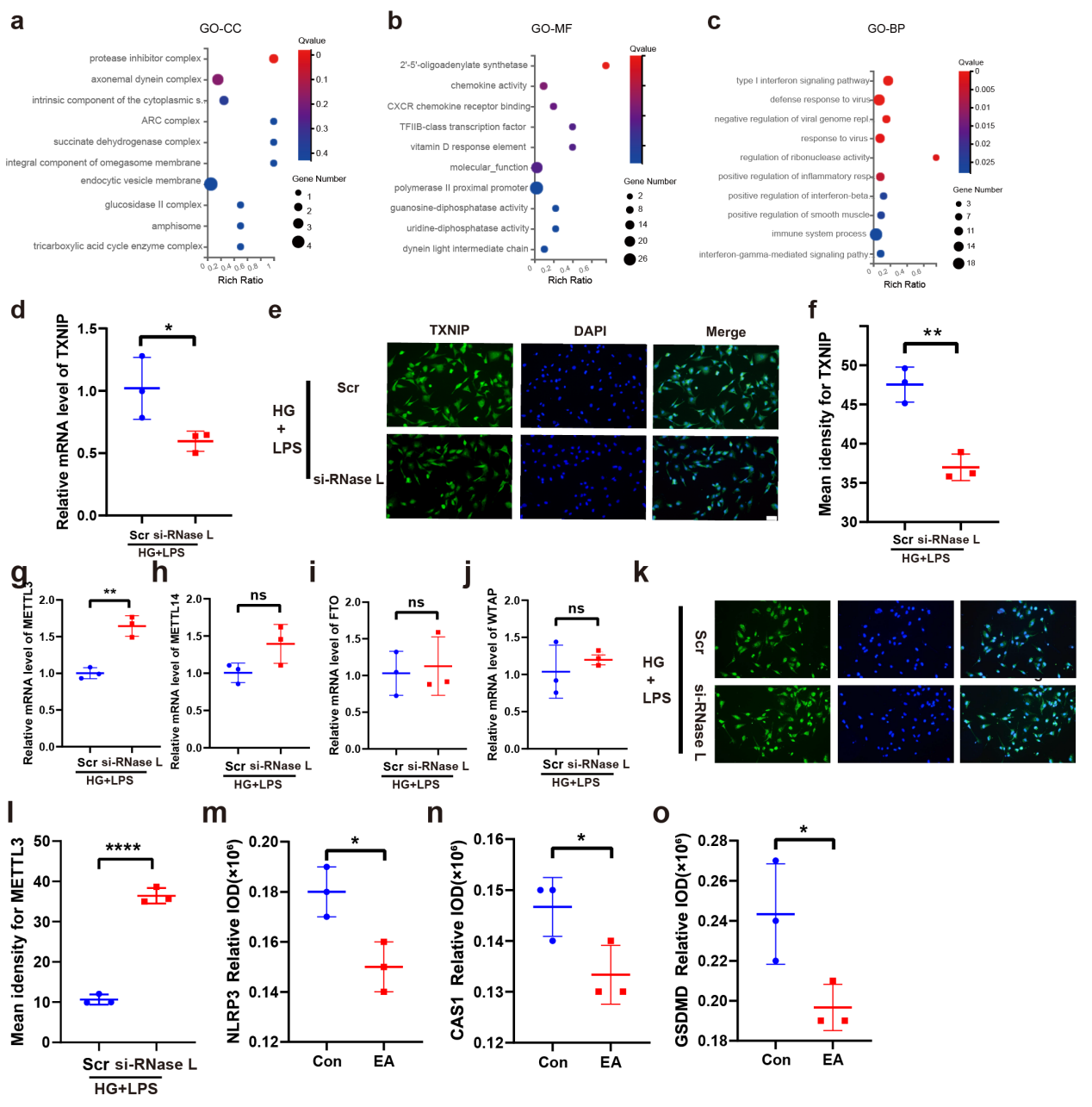


Figure S1. Supplementary non-critical results. Gene ontology (GO) enrichment analysis in the knockdown of RNase L in HUVECs treated with LPS under high glucose by RNA-seq (a-c). qPCR and IF assays of TXNIP expression (d-f). qPCR assays of four key methylation regulatory genes—METTL3, METTL14, FTO and WTAP (g-j). IF assays of METTL3 (k-l). Immunofluorescence semi-quantitative analysis of NLRP3, caspase-1 and GSDMD (m-o). The results are presented as the mean ± SEM, *p < 0.05, **p < 0.01, ****p < 0.0001. Each experiment was replicated for thrice.


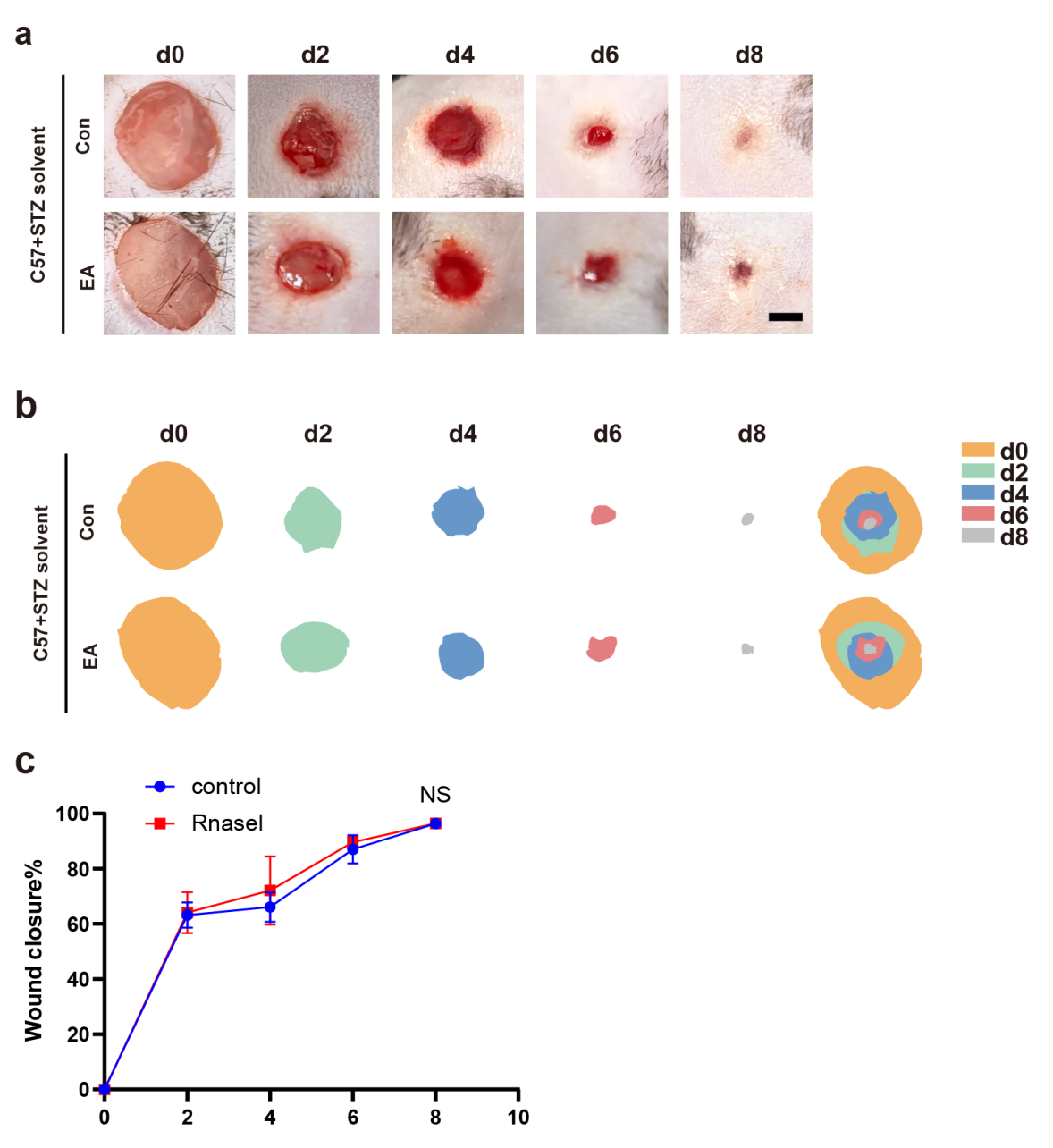


Figure S2. Effects of EA administered by gavage on wounds in normal mice. Representative gross images of wounds in mice (a). Illustrations depicting the wound healing process in A (b). Wound healing rates in each group at different time points (c). The number of mice used in this experiment was two per group, and two identical wounds were created on each mouse.

Table S1. The sequences of primer pairs used in qPCR.

| **Gene name** | **Direction** | **Sequences** |
| --- | --- | --- |
| Human OAS1 | Forward (5’–3’) | TGTCCAAGGTGGTAAAGGGTG |
|  | Reverse (3’–5’) | CCGGCGATTTAACTGATCCTG |
| Human OAS2 | Forward (5’–3’) | ACGTGACATCCTCGATAAAACTG |
|  | Reverse (3’–5’) | GAACCCATCAAGGGACTTCTG |
| Human OAS3 | Forward (5’–3’) | TCCGCCTGACATCCGTAGA |
|  | Reverse (3’–5’) | AGACTTGTGGCTTGGGTTTGA |
| Human RNase L | Forward (5’–3’) | GTAAACGCCTGTGACAATATGGG |
|  | Reverse (3’–5’) | AGATGCGTAATAGCCTCCACAT |
| Human FTO | Forward (5’–3’) | GGATGCTGTGCCATTGTGTATGTC |
|  | Reverse (3’–5’) | TCCACTTCATCTTGTCCGTTGTAGG |
| Human METTL3 | Forward (5’–3’) | TGCTTGGTTGGTGTCAAAGGAAATC |
|  | Reverse (3’–5’) | TGCTTGGTTGGTGTCAAAGGAAATC |
| Human METTL14 | Forward (5’–3’) | TGCTTGGTTGGTGTCAAAGGAAATC |
|  | Reverse (3’–5’) | TTGGAGCAGAGGTATCATAGGAAGC |
| Human WTAP | Forward (5’–3’) | AATCCAGTACCTCAAGCAAGTCCAG |
|  | Reverse (3’–5’) | AGGCGTAAACTTCCAGGCACTC |
| Human TXNIP | Forward (5’–3’) | CAGCAGTGCAAACAGACTTCGG |
|  | Reverse (3’–5’) | CAGCAGTGCAAACAGACTTCGG |
| Mouse TXNIP | Forward (5’–3’) | CTACTGATTGCCACCCATCTT |
|  | Reverse (3’–5’) | CTACTGATTGCCACCCATCTT |

Table S2. RNA quality

| Sample | Concentration (ng/μL) | Volume (μL) | Quality  (μg) | RIN/RQN | 28S/ 18S |
| --- | --- | --- | --- | --- | --- |
| HUVEC_NC1 | 95 | 25 | 2.38 | 9.9 | 1.3 |
| HUVEC_NC2 | 155 | 25 | 3.88 | 9.8 | 1.4 |
| HUVEC_NC3 | 155 | 25 | 3.88 | 9.8 | 1.9 |
| HUVEC_siRNA1 | 160 | 25 | 4 | 10.0 | 2.0 |
| HUVEC_siRNA2 | 130 | 25 | 3.25 | 9.7 | 1.7 |
| HUVEC_siRNA3 | 120 | 23 | 2.76 | 9.8 | 1.6 |


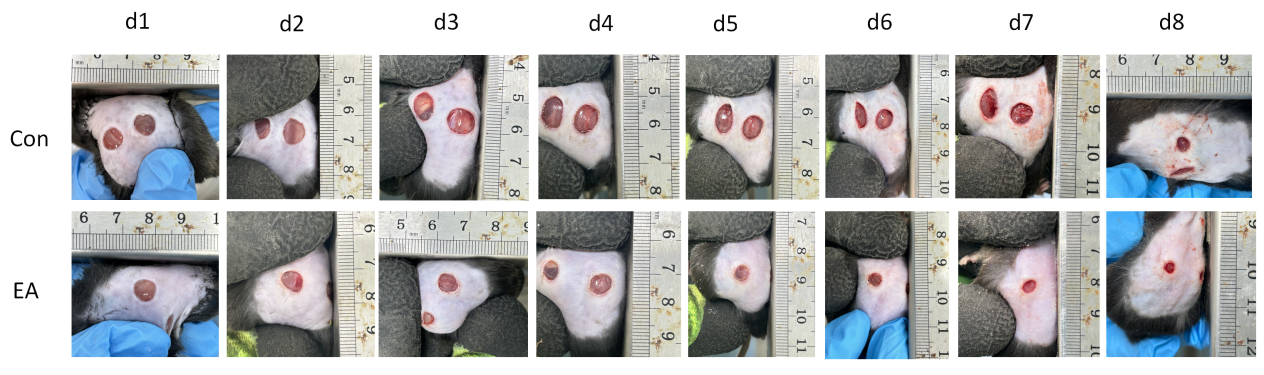


Figure S3. Wound photograph with scale.
